# Collective and synchronous dynamics of photonic spiking neurons

**DOI:** 10.1038/s41467-021-22576-4

**Published:** 2021-04-23

**Authors:** Takahiro Inagaki, Kensuke Inaba, Timothée Leleu, Toshimori Honjo, Takuya Ikuta, Koji Enbutsu, Takeshi Umeki, Ryoichi Kasahara, Kazuyuki Aihara, Hiroki Takesue

**Affiliations:** 1 NTT Basic Research Laboratories, NTT Corporation, 3-1 Morinosato Wakamiya, Atsugi, Kanagawa 243-0198 Japan; 2 Institute of Industrial Science, The University of Tokyo, 4-6-1, Komaba, Meguro-ku, Tokyo, 153-8505 Japan; 3 International Research Center for Neurointelligence, The University of Tokyo Institute for Advanced Study, The University of Tokyo, 7-3-1 Hongo, Bunkyo-ku, Tokyo, 113-0033 Japan; 4 NTT Device Technology Laboratories, NTT Corporation, 3-1 Morinosato Wakamiya, Atsugi, Kanagawa 243-0198 Japan

**Keywords:** Computational neuroscience, Nonlinear optics, Statistical physics, thermodynamics and nonlinear dynamics

## Abstract

Nonlinear dynamics of spiking neural networks have recently attracted much interest as an approach to understand possible information processing in the brain and apply it to artificial intelligence. Since information can be processed by collective spiking dynamics of neurons, the fine control of spiking dynamics is desirable for neuromorphic devices. Here we show that photonic spiking neurons implemented with paired nonlinear optical oscillators can be controlled to generate two modes of bio-realistic spiking dynamics by changing optical-pump amplitude. When the photonic neurons are coupled in a network, the interaction between them induces an effective change in the pump amplitude depending on the order parameter that characterizes synchronization. The experimental results show that the effective change causes spontaneous modification of the spiking modes and firing rates of clustered neurons, and such collective dynamics can be utilized to realize efficient heuristics for solving NP-hard combinatorial optimization problems.

## Abstract (web summary)

Neuromorphic devices take inspiration from spiking dynamics of neurons in the brain. Here, the authors demonstrate synchronized spiking dynamics in 240 photonic artificial neurons, each of which is implemented with a pair of antisymmetrically coupled degenerate optical parametric oscillators.

## Introduction

Specialized hardware that performs brain-inspired information processing has achieved significant success in the fields of machine learning and artificial intelligence^1–4^. To provide more biologically realistic functions with artificial systems^5,6^, various neuromorphic devices based on spiking neural network (SNN) models have been developed^7–10^. Neurons communicate with nerve impulses, called spikes or action potentials, and synchronization of the spikes can be useful for signal processing performed in the brain^11–14^. The nonlinear properties of optical oscillators have been expected to be suitable for fast and energy-efficient implementations of spiking neurons^15–22^, however, the photonic devices proposed so far have been generally limited in terms of the diversity of their spiking dynamics. Since most nervous systems are constructed with various types of neurons, the collective dynamics of different spiking modes can be important factors in building neuromorphic devices. Recently, various nonlinear dynamics of coupled parametric oscillators, which can be utilized for artificial spiking neurons, have been proposed^23,24^, and an effect of collective dynamics on the synchronization of the spikes has been demonstrated with an array of vertical cavity surface emitting lasers^25^.

In the present study, we demonstrated that a photonic artificial neuron can generate two different bio-realistic spiking modes that are changed spontaneously as a result of the synchronization within clusters of neurons. The artificial neuron was implemented with anti-symmetrically coupled degenerate optical parametric oscillators (DOPOs). The nonlinearity and phase bistability of the DOPOs were used to generate two spiking modes of class-I (saddle-node bifurcation) and class-II (Andronov-Hopf bifurcation) neurons that had been originally classified by A.L. Hodgkin^26^ and characterized in terms of different bifurcation structures^27,28^. Although it was shown that some neuron models can generate both class-I and class-II spiking modes depending on the values of the model parameters^27–32^, the spiking modes of component neurons in spiking neural networks are usually fixed in advance. The spiking mode of our proposed photonic neuron, on the other hand, can change due to collective and synchronous dynamics of the network for spontaneous information processing because the spiking dynamics can be controlled by tuning optical-pump amplitudes of the DOPOs. Network experiments with 240 DOPO neurons revealed that input signals from the correlating neurons can induce an effective change in the pump amplitude. The effective change depends on the increase in the order parameter of synchronization, and it causes spontaneous changes in spiking modes and firing rates of the networked neurons. The experimental results showed that the self-tuning effect of collective spiking dynamics can be utilized for solving combinatorial optimization problems by using methods related to self-organized criticality.

## Results

### Artificial spiking neuron with coupled DOPOs

**Fig. 1:**
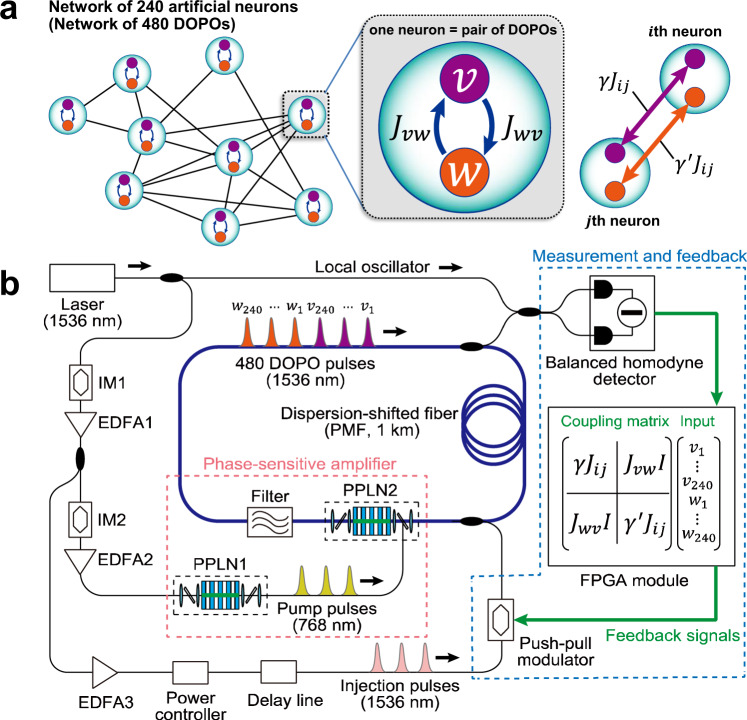
Experimental setup of a DOPO neural network. **a** 240-node artificial neural network composed of 480 DOPOs with antisymmetric couplings (${J}_{{vw}}=-{J}_{{wv}}$). **b** Schematic diagram based on time-domain multiplexing in a 1-km fiber-ring cavity. PPLN, periodically poled lithium-niobate; PMF, polarization-maintaining fiber; IM, intensity modulator; EDFA, erbium-doped-fiber amplifier; FPGA, field-programmable gate array.

In this study, a photonic spiking neuronal network was developed by utilizing a network of DOPO pulses in a fiber-ring cavity, which has been used for simulating an Ising spin network and solving combinatorial optimization problems^33–37^. The DOPO pulse is generated by a phase-sensitive amplifier (PSA) with a ${\chi }_{2}$ nonlinear material in the cavity^38–40^. Because degenerate parametric amplification is phase sensitive, the optical phase of each DOPO pulse takes only 0 or π relative to the pump pulse above the threshold of the cavity, and the optical amplitude of the bistable phase states can represent the positive and negative membrane potentials in the spiking neuron with sign-inversion symmetry; namely, the change in the sign of optical amplitude does not change the neuron properties. As shown in Fig. 1a, the spiking dynamics of each neuron are implemented by using a pair of coupled DOPO pulses (called $v$- and $w$-DOPOs) with respective coupling coefficients ${J}_{{vw}}$ and ${J}_{{wv}}$. The *i*th neuron in a DOPO neural network is modelled as

1 $$\frac{d{v}_{i}}{{dt}}=(-1+{p}_{i}){v}_{i}-{v}_{i}^{3}+{J}_{{vw}}{w}_{i}+\gamma \mathop{\sum}\limits_{j}\,{J}_{{ij}}{v}_{j}+{I}_{{\rm{ext}}}$$

2 $$\frac{d{w}_{i}}{{dt}}=(-1+{p}_{i}){w}_{i}-{w}_{i}^{3}+{J}_{{wv}}{v}_{i}+{\gamma }^{{\prime} }\mathop{\sum}\limits_{j}\,{J}_{{ij}}{w}_{j}$$

where ${v}_{i}$ and ${w}_{i}$ represent in-phase components of DOPO amplitudes, and ${p}_{i}$ and $I_{\mathrm{ext}}$ are optical amplitude of the pump pulse and an external-bias term, respectively. Quadrature components of DOPOs become negligible above the threshold due to the PSA^41–43^. Matrix $J_{ij}$ describes synaptic connections between the *i*th and *j*th DOPO neurons, and $\gamma$ (${\gamma}^{\prime}$) is a scaling factor of coupling strength for the $v$- ($w$-) DOPOs (see Supplementary Notes 1 and 2 for more details).

A schematic diagram of the networked DOPO neurons is shown in Fig. 1b. A network of 512 DOPO pulses based on time-domain multiplexing in a 1-km fiber cavity and opto-electronic feedback system was developed^35^. A periodically poled lithium-niobate (PPLN) waveguide module and an optical band-pass filter were placed in the fiber cavity as a PSA. The continuous wave from a laser with a wavelength of 1536 nm was modulated by a lithium-niobate intensity modulator (IM1) into sequential pulses with 60-ps width and 1-GHz repetition frequency. The sequential pulses were amplified by erbium-doped fiber amplifiers and converted into 768-nm pump pulses by second harmonic generation (SHG) in the first PPLN waveguide (PPLN1). The pump pulses were converted into signal and idler waves by parametric down-conversion (PDC) in the second PPLN waveguide (PPLN2) in the 1-km fiber ring cavity. An optical band-pass filter with center wavelength of 1536 nm and passband width of 13 GHz was set behind PPLN2 so that the transmitted light could satisfy the degenerated condition of the signal and idler waves. As a result of the interference of the degenerated signal and idler waves, in which the 0 or π phase component relative to the pump phase was amplified most efficiently^44^, phase-sensitive amplification could be obtained. When pumping of the PSA was started, quadrature squeezed noise pulses were generated by spontaneous parametric down-conversion in the PPLN waveguide. The noise pulses were amplified in each cavity circulation by the PSA, and that amplification led to the formation of time-domain multiplexed DOPO pulses. Since the pump pulse interval was 1 ns and the round-trip time of the 1-km fiber cavity was 5 μs, the cavity could accommodate more than 5000 DOPOs, 512 of which were used for this experiment.

Arbitrary all-to-all connectivity between the 512 DOPOs was implemented by using a measurement-and-feedback (MFB) scheme^45^. During each cavity circulation, a portion of each DOPO pulse was extracted with a 9:1 coupler, and the in-phase component was measured with a balanced homodyne detector (BHD). The local oscillator for the BHD was supplied from the continuous-wave laser, which was used for preparing the pump pulse. The measured signals were then input into a field-programmable gate array (FPGA) module. The feedback signal for each DOPO pulse was calculated by using the input signals and a 512 × 512 coupling matrix with 8-bit connection weight resolution. The calculated feedback signals were imposed on the optical pulses by using a push-pull modulator and injected into the cavity at times synchronized with the target DOPO pulses. By repeating this procedure, it was possible to connect the 512 DOPO pulses in different time slots arbitrarily. Thirty-two DOPOs were used as header pulses to monitor experimental conditions, and 480 DOPOs were assigned to simulate 240 DOPO neurons. The internal and external optical couplings of the 240 DOPO neurons could be controlled by changing the connection weights stored with the coupling matrix in the FPGA module.

### Control of spiking dynamics

**Fig. 2:**
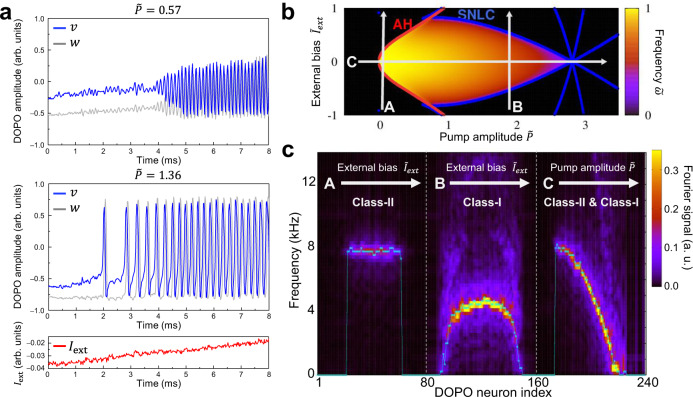
Class-I and II spiking modes of a DOPO neuron. **a** Time evolutions of $v$- and $w$-DOPOs (blue and gray lines) with constant pump amplitudes ($\widetilde{P}$ = 0.57 and 1.36) and an external bias increased linearly with time. **b** Phase diagram of the DOPO neuron in the parameter space of $P$ and ${I}_{{\rm{ext}}}$. The color map represents spiking frequencies calculated by numerical simulations. Red and blue dots obtained by linear stability analysis represent points where the Andronov–Hopf (AH) bifurcation and saddle-node bifurcation on a limit cycle (SNLC) occur, so they characterize class-II and class-I neurons, respectively. **c** Experimental results of tomographic measurement of spiking frequencies along lines A, B, and C in (**b**). The color map represents Fourier signals of time evolutions of DOPO amplitudes. Cyan points are firing rates estimated by adding up the number of spikes.

Spiking behavior of DOPO neurons was controlled by changing pump amplitude. Time evolutions of coupled $v$- and $w$-DOPOs (blue and gray lines, respectively) were observed as shown in Fig. 2a. Constant pump amplitudes and an external bias linearly increasing from a negative value were applied to the DOPO neuron with antisymmetric coupling (${J}_{{vw}}=-{J}_{{wv}}$). The following two parameters are defined: $P=\left(-1+p\right)$ and ${\omega }_{0}=\sqrt{-{J}_{{vw}}{J}_{{wv}}}$, where ${\omega }_{0}$ is natural spiking frequency at $P=0$. Dimensionless notations are given as ${\widetilde{I}}_{{\rm{ext}}}\equiv {I}_{{\rm{ext}}}/{\omega }_{0}^{3/2}$ for bias and $\widetilde{X}\equiv X/{\omega }_{0}$ with $X=P,\gamma ,$ and so on. For large $\widetilde{P}$ (bottom panel of the figure), the interspike intervals gradually decrease from a very large value, meaning that firing rates gradually increase with increasing bias. For small $\widetilde{P}$ (top panel), the interspike intervals and firing rates hardly change, while amplitude gradually increase with increasing bias. These two kinds of behavior are very similar to those of the class-I and class-II neurons^26,28^.

Spiking behavior of a single DOPO neuron was investigated by tuning pump amplitude $P$ and external bias ${I}_{{\rm{ext}}}$ with both $P$ and ${I}_{{\rm{ext}}}$ constant over time. Moreover, the model described by Eqs. (1) and (2) was validated by comparing experimental results with numerical simulations (see Supplementary Note 1). Change in spiking frequency $\widetilde{\omega }$ as a function of $\widetilde{P}$ and ${\widetilde{I}}_{{\rm{ext}}}$ calculated by those numerical simulations is plotted in Fig. 2b. As shown in Fig. 2c, the spiking frequencies of DOPO neurons were experimentally measured with different operating parameters along three lines (A, B, and C) in Fig. 2b. Our experimental setup could simulate up to 240 DOPO neurons simultaneously, and 80 neurons were assigned for obtaining data points corresponding to each line. Both the experimental measurements and simulations clearly show a sudden rise and fall of $\omega$ for line (A) and gradual increase and decrease of $\omega$ for line (B), characterizing the class-II and class-I neurons, respectively. These results show that the spiking mode can be switched between the classes II and I by tuning pump amplitude $P$. These behaviors can be explained by the saddle-node bifurcation on a limit cycle (SNLC) (corresponding to class-I neuron) and the Andronov-Hopf (AH) bifurcation (class-II neuron) (see Supplementary Note 2). The neuron classes could be controlled by changing $P$ with ${I}_{{\rm{ext}}}=0$ on line (C). When $P$ was changed from negative to positive, firing rate increases suddenly (AH) and then gradually decreases to zero (SNLC). Change in firing rate at ${I}_{{\rm{ext}}}=0$ is approximately predicted as a function of $P$ as

3 $$\omega \left(P\right)={\omega }_{0}\sqrt{1-\frac{{P}^{2}}{8{\omega }_{0}^{2}}},$$

where $0\le \widetilde{P}\le \sqrt{8}$ (see Method). The experimental result agrees well with the prediction of this function, and it was confirmed that the spiking mode of the DOPO neuron can be seamlessly controllable between the class-I and class-II excitability.

### Spontaneous modification of collective dynamics

**Fig. 3:**
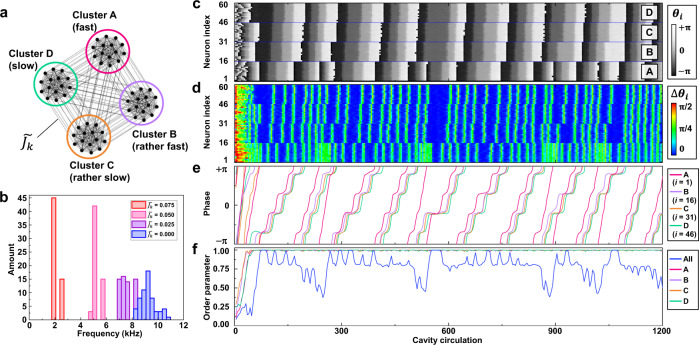
Synchronization experiment using clustered Kuramoto models. **a** Structure of DOPO-neuron network consisting of four clusters of 15 neurons. **b** Firing-rate distribution of 60 neurons. For ${J}_{k}=0$, the distribution is spread as designed on the basis of pump amplitude. **c** Time evolutions of phase ${\theta }_{i}$ of the *i*th DOPO neuron with coupling of $\widetilde{{J}_{k}}$ = 0.075, where ${\theta }_{i}$ is an angle defined in the $v$-$w$ plane of the coupled DOPOs. **d** Phase change $\triangle {\theta }_{i}$ per four cavity circulations. **e** Phase ${\theta }_{i}$of a sampled neuron in each cluster. **f** Order parameter $r$ for each cluster and for all 60 neurons. Synchronization can be characterized by $r\sim 1$.

Synchronization phenomenon of networked DOPO neurons, which is an essential factor in signal processing of a SNN, was investigated next. Networks of 60 DOPO neurons were constructed as depicted in Fig. 3a. In each network, 15 neurons form an all-to-all connected cluster (like the Kuramoto model^46,47^; also see Method), and four such clusters are sparsely connected. This network structure was encoded into connections of both $v$- and $w$-DOPOs (i.e., $\gamma ={\gamma }^{{\prime} }\equiv {J}_{k}$). Since 240 neurons could be implemented in our experimental setup, four independent sets of such ensembles, consisting of 60 DOPO neurons with different coupling strengths ($\widetilde{{J}_{k}}$ = 0, 0.025, 0.05, and 0.075), were measured at the same time under similar experimental conditions as shown in Supplementary Fig. 6. We set ${I}_{{\rm{ext}}}=0$ and used uniform $i$-independent coupling $|{J}_{{vw}}|=\left|{J}_{{wv}}\right|$. Then, *i*-dependence of the pump amplitude *P*_*i*_ was tuned to control the distribution of ${\omega }_{i}\left({P}_{i}\right)$, and the ${P}_{i}$ were assigned to four 15-neuron clusters labeled A to D in descending order of firing rate. The distribution of firing rates of the 60 DOPO neurons is shown in Fig. 3b. The distribution of intrinsic firing rates was measured without coupling ($\widetilde{{J}_{k}}=0$), and the firing rates are widely spread according to applied ${P}_{i}$. With weak coupling at $\widetilde{{J}_{k}}$ = 0.025, the distribution of firing rates is shown as four uniform frequencies, suggesting that coupled DOPO neurons reach obvious synchronization in each 15-neuron cluster; however, the four clusters are unsynchronized. As ${J}_{k}$ increases, mean and variance of the firing rate decreases. Time evolutions of DOPO neurons with coupling at ${\widetilde{J_{k}}}$ = 0.075 are shown in Fig. 3c, where ${\theta }_{i}\equiv {\mathrm{arg}}\left({v}_{i}+i{w}_{i}\right)$ is a phase defined on the $v$ - $w$ plane of amplitudes of paired DOPOs. Phase change ${\triangle \theta }_{i}$ in each of the four cavity circulations is shown in Fig. 3d. Phases of the sampled neurons in the four clusters are shown in Fig. 3e. Order parameter $r$, defined as $r=\left|\frac{1}{N}\mathop{\sum}\limits_{j}{e}^{i{\theta }_{j}}\right|$, for each cluster (*N* = 15) and for all neurons (*N* = 60) was evaluated as shown in Fig. 3f. By increasing coupling strength to $\widetilde{{J}_{k}}$ = 0.075, the four clusters became intermittently synchronized, and the order parameter occasionally reached $r\sim 1$. Additionally, a similar experiment was performed when the 60 neurons form a single cluster with all-to-all connection. As shown in Supplementary Fig. 7, phase locking of all neurons was observed by increasing coupling strength $\widetilde{{J}_{k}}$ while keeping the order parameter high value $r\sim 1$.

The above-described behavior of the order parameter can be understood by the Kuramoto model^46,47^. However, it should be noted that firing rates are significantly decreased from their original values as ${J}_{k}$ increases as shown in Fig. 3b, and that behavior differ from the standard behavior expressed by the Kuramoto model. Further analysis suggests that the change in firing rate is induced by an effective change in the spiking mode of synchronized DOPO neurons as an ensemble. The dynamics of the networked DOPO neurons defined by Eqs. (1) and (2) can be rewritten with $\sqrt{{R}_{i}}{e}^{i{\theta }_{i}}\equiv {v}_{i}+i{w}_{i}$ as

4 $$\frac{d{\theta }_{i}}{{dt}}={\omega }_{0}-{J}_{k}\mathop{\sum}\limits_{j\ne i}{\varepsilon }_{{ij}}{\rm{sin }}({\theta }_{i}{-\theta }_{j})+\frac{{R}_{i}}{4}{\rm{sin }}4{\theta }_{i}$$

5 $$\frac{d{R}_{i}}{{dt}}=2{P}_{i}{R}_{i}+{2J}_{k}{R}_{i}\mathop{\sum}\limits_{j\ne i}{\varepsilon }_{{ij}}{\rm{cos }}({\theta }_{i}{-\theta }_{j})-\frac{{R}_{i}^{2}}{2}\left({\rm{cos }}4{\theta }_{i}+3\right),$$

where ${\varepsilon }_{{ij}}=\sqrt{{R}_{j}{/R}_{i}}$. The quadrature components of the DOPOs are neglected here. Equation (4) is analogous to the standard Kuramoto model (see Method). Near the limit of $P\to +0$ where ${R}_{i}\to 0$, ${R}_{i}$ terms can be neglected, and a simple Kuramoto model can be obtained with $\frac{d{\theta }_{i}}{{dt}}=\omega \left({P}_{i}\right)-{J}_{k}\mathop{\sum}\limits_{j\ne i}{\rm{sin }}({\theta }_{i}{-\theta }_{j})$. Here, each class-II DOPO neuron is a simple oscillator with angle frequency $\omega \left(P\right)$ that slightly depends on ${P}_{{\rm{i}}}$ as given in Eq. (3) around $P=0$. Away from $P\sim 0,$ the term ${R}_{i}{\rm{sin }}4{\theta }_{i}$ in Eq. (5) suggests the class change of neurons from the class-II to the class-I. Indeed, each oscillator obeys the simple SNLC type equation $\frac{d\theta }{{dt}}={\omega }_{0}+\frac{P}{\sqrt{8}}\left(-1+8{\theta }^{2}\right)$ near the saddle point at around $\theta =\frac{1}{4}(\frac{\pi }{2}+2n\pi )$ with $R\cong \sqrt{2}P$ in the case without interaction ($\widetilde{{J}_{k}}=0$) (see Supplementary Note 2). By taking account of the finite interactions effectively, Eq. (5) indicates that the pump term should be renormalized as ${P}_{i}^{{\prime} } = {P}_{i} + {J}_{k}\mathop{\sum}\limits_{j\ne i} {\varepsilon }_{{ij}}\, {\mathrm{cos}} ({\theta}_{i} {-\theta }_{j})$, and the second term on the right-hand side can be reduced to $r(N-1){J}_{k}$ under the approximation ${\varepsilon }_{{ij}}\sim 1$. This result suggests that the synchronization with a large order parameter $(r\sim 1)$ increases the pump term effectively and causes the spontaneous change in the spiking mode from class-II to class-I.

The initial part of the dynamics in Fig. 3e shows characteristics of the class-II neuron, a linearly increasing ${\theta }_{i}$ at a gradient of $d{\theta }_{i}/{dt}$ $\sim {\omega }_{0}$, where Fig. 3f shows the order parameter is not yet increased (*r* < 1). On the other hand, the latter part of the dynamics, where $r\sim 1$, shows the features of a class-I neuron; e.g., the latter part of Fig. 3e shows stepwise structures indicating that the neuron repeatedly stays on near unstable stationary points $(d{\theta }_{i}/{dt}\sim 0)$ and escapes from those points. Note that the timing of the escape is flexibly tuned (see Fig. 3d, in which the width of the blue areas with $\triangle {\theta }_{i}\sim 0$ frequently changes). It is also shown in Fig. 3e that during a long escape time from the neighborhood of unstable stationary points, the order parameter $r$ tends to increase up to 1, as shown in Fig. 3f, suggesting that the timing of the spikes is tuned spontaneously to increase the order parameter. For the unsynchronized ensemble, on the other hand, the effective change in the pump term is suppressed with small $r$, and the spiking mode reverts to class-II. Since the spiking dynamics of the class-II neuron has no stationary point, the tuning of the spike timing is suppressed. Consequently, the self-tuning effect of collective spiking dynamics of clustered neurons assists the overall synchronization, even though each cluster has significantly different firing rates.

### Combinatorial optimization using self-tuning dynamics

**Fig. 4:**
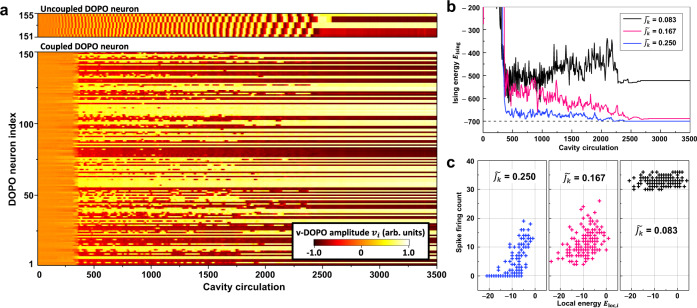
Spiking dynamics when solving a 150-node Ising problem. **a** Time evolutions of $v$-DOPO amplitudes with couplings of $\widetilde{{J}_{k}}$ = 0.250 (lower) and without coupling (top). Pump amplitudes increase linearly with time. **b** Time evolutions of Ising energy for $\widetilde{{J}_{k}}$ = 0.083, 0.167, and 0.250. Strong couplings give the best-known solution. **c** Relationship between local energy and firing rate. Firing rate and local energy are positively correlated with strong couplings, suggesting that energetically unstable neurons have high firing rate due to the self-tuning effect of the spiking mode.

To understand the effect of the above-described spontaneous change in firing rates of local clusters on the whole network dynamics, an analogy with a complex frustrated spin system, namely, the Ising model^48^, is considered as follows. The state of the Ising model is described in terms of Ising energy given by ${E}_{{\rm{Ising}}}=-\mathop{\sum}\limits_{i < j}{J}_{{ij}}{\sigma }_{i}{\sigma }_{j}$, where ${\sigma }_{i}=\{-1,+1\}$ denotes the *i*th Ising spin state, and ${J}_{ij}$ is a matrix representing symmetric spin–spin interaction between the *i*th and *j*th spins. The Ising spin state can be represented by the binary phase state of the $v$-DOPO. Spin–spin interaction ${J}_{{ij}}$ is implemented only for synaptic connections between $v$-DOPOs ($\gamma =-{J}_{k},{\gamma }^{{\prime} }=0$). External bias ${I}_{{\rm{ext}}}$ is set to zero. Of particular interest is relaxation of the DOPO SNN to configurations with lower Ising energy, which can be used to solve many combinatorial optimization problems. As the first benchmark problem, the following instance was solved: a highly frustrated network of 150 Ising spins coupled by symmetric connections with edge density of 50%. This instance has been investigated by using various algorithms, such as the one used in a spin-glass server^49^, and physical systems based on networked DOPOs, such as a coherent Ising machine (CIM)^50^. The best-known solution of the instance has ${E}_{{\rm{Ising}}}$ of $-$700. Note that the probability of reaching the best-known solution by using the CIM was 0.8% with computation time of 5 ms (1000 cavity circulations), and we confirmed that the instance is not an easy instance based on the optimisation simplicity criterion (OSC) proposed by Kalinin, et al.^51^. The time-dependent and node-independent pump amplitude ${P}_{i}={P}_{0}(t)$ was applied to the DOPOs, and the amplitude linearly increased with time. Time evolution of measured $v$-DOPO amplitudes with coupling strength $\widetilde{{J}_{k}}$ = 0.250 is shown in Fig. 4a. As a reference, the dynamics of uncoupled DOPO neurons are shown in the top panel. This figure suggests that the firing rate gradually decreases with time, and the spiking dynamics terminate at the end of calculation because ${P}_{0}(t)$ finally reaches a value exceeding the spiking region. When the network is connected (see lower panel), DOPO neurons show highly irregular and complex spiking behavior. Time evolutions of the Ising energy with various coupling strength ($\widetilde{{J}_{k}}$ = 0.083, 0.167, and 0.250) are shown in Fig. 4b. Lower-energy solutions are found with higher coupling strengths at $\widetilde{{J}_{k}}=\,$0.250, and the best-known solution is obtained with success probability of 11% and computation time of 17.5 ms (3500 cavity circulations). In terms of the time-to-solution, the performance of the DOPO spiking neural network is better than that of the CIM for this instance (see Supplementary Note 7 for more details). To understand the dependence of the spiking dynamics on coupling strength, the relationship between the total firing count of each DOPO neuron and local energy ${E}_{{\rm{loc}},{i}}$ of the final solution is shown in Fig. 4c. Local energy is defined as ${E}_{{\rm{loc}},{i}}=-\mathop{\sum}\limits_{j}{J}_{{ij}}{\sigma }_{i}{\sigma }_{j}$, which is related to Ising energy by ${E}_{{\rm{Ising}}}=\frac{1}{2}{\sum }_{i}{E}_{{\rm{loc}},i}$. With increasing coupling strength, a positive correlation between firing count and local energy appears. As clarified in the above discussion, synchronization of DOPO neurons causes firing rates to change. The order parameter can be related to local energy ${E}_{{\rm{loc}},{i}}$ by using certain approximations (see Supplementary Note 7); thus, renormalized pump amplitude can be rewritten as ${P}_{i}={P}_{0}(t)-\frac{1}{2}{J}_{k}{E}_{{\rm{loc}},{i}}(t)$. This result means that DOPO neurons with higher ${E}_{{\rm{loc}},{i}}$ (energetically unstable nodes) show higher firing rates and those with lower ${E}_{{\rm{loc}},{i}}$ (energetically stable nodes) show lower firing rates. Consequently, the DOPO neural network spontaneously tries frequently to flip Ising spins primarily on energetically unstable nodes, and such a selective spin-flip mechanism should be a key factor in accelerating the relaxation to lower energy states. The dependence of the spin-flip frequency with corresponding local energy is reminiscent of the state-of-the-art algorithms for combinatorial optimization such as extremal optimization^52^ and methods related to self-organized criticality^53^.

## Discussion

It was confirmed that the spiking dynamics of the DOPO neurons can be controlled from class-II to class-I neuronal mode by increasing optical-pump amplitude, and firing rate can be modulated dynamically by the crossover of different spiking modes. This flexible controllability of spiking modes induces a self-tuning effect in the collective dynamics of the DOPO neurons. Because the pump amplitudes of clustered DOPO neurons can be approximately renormalized as ${P}_{i}^{{\prime} }={P}_{i}+r(N-1){J}_{k}$, the pump amplitude can be effectively increased as the order parameter $r$ of synchronization is increased. Our experimental results show that the firing rate of neuron is modulated according to the order parameter. Such spontaneous modification of the collective dynamics assisted the synchronization even though neurons have significantly different firing rates. Additionally, the self-tuning effect of firing rate was utilized to improve the optimization process of the Ising spin network. Since the renormalized pump amplitude can be rewritten as ${P}_{i}^{{\prime} }={P}_{i}-\frac{1}{2}{J}_{k}{E}_{{\rm{loc}},{i}}$ in the case of the antiferromagnetic spin network, the firing rate of neuron is modulated according to local energy ${E}_{{\rm{loc}},{i}}$. Firing-rate selectivity for the local energy might be an effective way to find global lower-energy solutions of the Ising model. The present DOPO neural network inherently includes such a dynamical optimization process thanks to the self-tuning effect of the collective spiking dynamics.

In our experimental setup, the $v$-$w$ coupling and network of DOPO neurons were implemented with the measurement-and-feedback scheme based on the FPGA module and thus the computation speed can be limited by the matrix calculation performed by the FPGA module (see Supplementary Note 7). We expect that the time scale of spiking frequency ${\omega }_{0}$ has the potential to become shorter by relying on 10-GHz-repetition rate lasers and replacing the $v$-$w$ coupling based on the FPGA module by direct optical coupling with an integrated photonics platform. Fortunately, flexible optical couplings using integrated interferometers in a waveguide module^54,55^ and spatial light modulators^56,57^ have been proposed for advanced photonic Ising machines^58,59^. These activities can greatly support faster photonic implementation of the artificial spiking neuron with coupled DOPOs. On the other hand, the current scheme using the FPGA module is very effective for implementing dense networks like the Kuramoto model since the optical coupling can suffer from limitation of connectivity due to spatial restriction and resolution of the physical system. Thus, our final goal will be to explore the best balance of the combination of photonic neuromorphic elements and the measurement-and-feedback scheme, mimicking the combination of neurons and synapse connections in a real neural system.

## Method

### Analysis of linear stability

Hereafter, the bifurcation of a single neuron is discussed without consideration of interneuron couplings and external fields ($\gamma ={\gamma }^{{\prime} }={I}_{{\rm{ext}}}=0$). It is assumed that the $i$-dependence can be neglected in the notations. Analysis of linear stability based on Eqs. (1) and (2) explains what kinds of bifurcations appear. A linearized form around an equilibrium point $$\left(\begin{array}{c}v\\ w\end{array}\right)=\left(\begin{array}{c}{v}_{e}\\ {w}_{e}\end{array}\right)$$ is given by $$\frac{d}{{dt}}\left(\begin{array}{c}v\\ w\end{array}\right)=M\left(\begin{array}{c}v\\ w\end{array}\right)$$ with $$M=\left(P-3{v}_{e}^{2} -{\omega }_{0}\\ {\omega }_{0} \,P-3{w}_{e}^{2}\right)$$. For small $P( > 0)$, equilibrium found at $$\left(\begin{array}{c}{v}_{e}\\ {w}_{e}\end{array}\right)=\left(\begin{array}{c}0\\ 0\end{array}\right)$$ has eigenvalues of $M$ given by $\lambda =P\pm i{\omega }_{0}$, suggesting an AH bifurcation. For large $P$, equilibriums can be found at the tangency points of nullclines around $$\left(\begin{array}{c}{v}_{e}\\ {w}_{e}\end{array}\right)\sim \left(\begin{array}{c}\pm \sqrt{P/3}\\ \mp 2P\sqrt{P}/3{\omega }_{0}\end{array}\right)$$. Around such equilibriums, two of $\lambda$ can be real and positive values that characterize the SNLC bifurcation. Numerical calculations can precisely estimate equilibriums and corresponding ${\rm{c}}$, and the sets where AH or SNLC bifurcations occur can then be evaluated, as shown in Fig. 2b. In addition, by simulating the spiking dynamics based on Eqs. (1) and (2), it is also possible to calculate spiking frequency. These two kinds of calculations are consistent with each other. See Supplementary Note 1 and 2 for more mathematical explanations.

### Spiking frequency

From Eqs. (4) and (5) (in which ${J}_{k}=0$ is set and $i$-dependence is neglected), spiking frequency $\omega \left(P\right)$ can be approximately obtained. $R$ can be roughly evaluated under the condition $\frac{{dR}}{{dt}}=0$ as $R\sim \frac{2P}{{\rm{\pi }}}{\int }_{-{\rm{\pi }}}^{{\rm{\pi }}}\frac{d\theta }{3+{\rm{cos }}4\theta }=\sqrt{2}P$, meaning that the number of photons ($\propto R={v}^{2}+{w}^{2}$) increases linearly with pump amplitude. From this relation $\frac{d\theta }{{dt}}={\omega }_{0}+\frac{P}{\sqrt{8}}{\rm{sin }}4\theta$ is obtained, and the period of the oscillator can be approximately evaluated as $T={\int }_{-{\rm{\pi }}}^{{\rm{\pi }}}\frac{d\theta }{{\omega }_{0}+\frac{P}{\sqrt{8}}{\rm{sin }}4\theta }=\frac{2{\rm{\pi }}}{\sqrt{{\omega }_{0}^{2}-{P}^{2}/8}}$. Finally, $\omega \left(P\right)$ in Eq. (3) is obtained. See Supplementary Note 3 for more detail about this calculation.

### Kuramoto model

The Kuramoto model, which provides a paradigm for understanding the mechanism of synchronization phenomena of coupled nonlinear oscillators^46,47^, is briefly introduced hereafter. The Kuramoto model for *N* nonlinear oscillators with all-to-all coupling with strength ${J}_{k}$ is given as

6 $$\frac{d{\theta }_{i}}{{dt}}={\omega }_{i}-{J}_{k}\mathop{\sum}\limits_{j}{\rm{sin }}({\theta }_{i}{-\theta }_{j})$$

where ${\theta }_{i}$ and ${\omega }_{i}$ are the phase and natural angular frequency of the *i*th nonlinear oscillator for *i* = 1, 2, …, *N*. Synchronization of coupled oscillators can be understood as a kind of phase transition characterized by order parameter $r$ defined as $r=\frac{1}{N}\mathop{\sum}\limits_{j}{e}^{i{\theta }_{j}}$. Under the assumption that ${\omega }_{i}$ has a distribution with variance ${\sigma }_{\omega }$, $r$ is zero for small ${J}_{k}/{\sigma }_{\omega }$ and becomes finite for large ${J}_{k}/{\sigma }_{\omega }$ (also see Supplementary Note 4). In our experiments, the distribution of ${\omega }_{i}$ was introduced by controlling pump amplitude ${P}_{i}$ through Eq. (3).

## Supplementary information

Supplementary Information

## Data Availability

The data that support the plots within this paper and other findings of this study are available from the corresponding authors upon reasonable request.

## Code availability

The modeling is described in the Supplementary information and the code is available from the corresponding authors upon reasonable request.
