# Supplementary material for: Collective and synchronous dynamics of photonic spiking neurons
Source: Nat Commun. 2021 Apr 23;12:2325. doi: 10.1038/s41467-021-22576-4 (PMC8065174; doi:10.1038/s41467-021-22576-4)
Supplement: Supplementary file 1 — Supplementary Information [file 41467_2021_22576_MOESM1_ESM.pdf]

Supplementary Information

- Collective and synchronous dynamics of photonic spiking neurons

Takahiro Inagaki, Kensuke Inaba, Timothée Leleu, Toshimori Honjo, Takuya Ikuta, Koji Enbutsu, Takeshi Umeki, Ryoichi Kasahara, Kazuyuki Aihara, and Hiroki Takesue

Supplementary Note 1 - *Numerical simulation*

The dynamics of a single DOPO neuron were investigated by numerical simulations based on the following coupled ordinary differential equations (ODEs):

$$\frac{dv}{dt} = -v + (1+P)v^* - |v|^2 v + J_{vw}\mathrm{Re}(w) + I_{ext}, \quad (1)$$

$$\frac{dw}{dt} = -w + (1+P)w^* - |w|^2 w + J_{wv}\mathrm{Re}(v), \quad (2)$$

where variables v and w are amplitudes of DOPOs called v- and w-DOPO, v^* and w^* are their complex conjugates, and $\mathrm{Re}(x)$ and $\mathrm{Im}(x)$ are in-phase and quadrature-phase components of DOPO amplitudes for $x = v, w$. Parameters P and I_{ext} are respectively optical-pump amplitude and an external bias term. J_{vw} and J_{wv} are connections between the v- and w-DOPOs, where these couplings are implemented by the measurement feedback method and is applied only to the in-phase component. Without coupling, each equation is the same as that describing the dynamics of a single DOPO[1-3]. For $P > 0$, the real components of v and w are amplified, while the imaginary ones are damped, so the latter components can be neglected. Note that our definition of optical-pump amplitude P is slightly modified from the definitions used in the previous papers, and the use of P

is convenient for discussing the spiking dynamics found in the real components of DOPO amplitudes v and w. The previous definition of optical-pump amplitude p is normalized by the threshold amplitude of an uncoupled DOPO, where oscillation occurs at $p = 1$. The present defection P is given by $p - 1$. Antisymmetric coupling with $J_{vw} = -J_{wv}$ (generally speaking, asymmetric coupling with $J_{vw}J_{wv} < 0$) induces energy transport between v and w and yields a stable limit cycle, which can be interpreted as a repetitive spiking process. Supplementary Equations 1 and 2 can be rewritten as

$$\frac{d\mathrm{Re}(v)}{dt} = P\mathrm{Re}(v) - |v|^2\mathrm{Re}(v) + J_{vw}\mathrm{Re}(w) + I_{ext} \ , \tag{3}$$

$$\frac{d\mathrm{Re}(w)}{dt} = P\mathrm{Re}(w) - |w|^2\mathrm{Re}(w) + J_{wv}\mathrm{Re}(v) \ , \tag{4}$$

$$\frac{d\mathrm{Im}(v)}{dt} = (-2 - P)\mathrm{Im}(v) - |v|^2\mathrm{Im}(v) \ , \tag{5}$$

$$\frac{d\mathrm{Im}(w)}{dt} = (-2 - P)\mathrm{Im}(w) - |w|^2\mathrm{Im}(w) \ , \tag{6}$$

where Supplementary Equations 3 and 4 correspond to Equations 1 and 2 in the main text.

Two parameters that characterize the properties of bifurcations (see also Supplementary Notes 2) are defined as basic frequency $\omega_0 \equiv \sqrt{-J_{vw}J_{wv}}$ and anisotropy of couplings between v- and w-DOPOs $\alpha \equiv \left|\frac{J_{wv}}{J_{vw}}\right|$, which are

controllable in the case of the present DOPO system. In the current study, the isotropic case $\alpha = 1$ is mainly considered. It is convenient to use ω_0 as the time unit in Supplementary Equations 1 and 2 so that the above equations can be rescaled to dimensionless forms by using $\tilde{v} = v/\sqrt{\omega_0}$, $\tilde{w} = w/\sqrt{\alpha\omega_0}$, $\tilde{P} = P/\omega_0$, $\tilde{I}_{ext} = I_{ext}/\sqrt{\omega_0}^3$, and $\tilde{t} = t\omega_0$. The dimensionless forms are given by $\frac{d\tilde{v}}{d\tilde{t}} = -\tilde{v} + (1+\tilde{P})\tilde{v}^* - |\tilde{v}|^2\tilde{v} \mp \mathrm{Re}(\tilde{w}) + \tilde{I}_{ext}$, and $\frac{d\tilde{w}}{d\tilde{t}} = -\tilde{w} + (1+\tilde{P})\tilde{w}^* - \alpha|\tilde{w}|^2\tilde{w} \pm \mathrm{Re}(\tilde{v})$.

The present numerical simulation takes into account the noise terms given by $\sqrt{|v|^2 + \frac{1}{2}}dW_{\mathrm{Re}(v)}$, $\sqrt{|v|^2 + \frac{1}{2}}dW_{\mathrm{Im}(v)}$, $\sqrt{|w|^2 + \frac{1}{2}}dW_{\mathrm{Re}(w)}$ and $\sqrt{|w|^2 + \frac{1}{2}}dW_{\mathrm{Im}(w)}$, where dW_x is the independent Gaussian noise for real and imaginary parts of v- and w-DOPOs[1-3]. Except for the following numerical simulation, these noise terms are neglected for simplicity and to focus on the mathematical study of the bifurcations.

Supplementary Figure 1 shows numerically simulated time series of DOPO amplitudes and the corresponding trajectories in the v-w space with the external bias $I_{ext} = 0$ or $\tilde{I}_{ext} = -0.3$ for $\tilde{P} = 0.3, 1.1, 1.8$ and 2.5. Numerical simulations used the Runge–Kutta method with parameters $\mathrm{dt} = 0.05$ and $dW_x \equiv 0.02 \times$ Gaussian noise. For comparison, trajectories

without noise terms ($dW_x = 0$) are also presented in the figure. A firing rate decreases as the pump amplitude increases, and qualitative features of the time series and the trajectories are also changed. For small P, a simple limit cycle is found at around $(\mathrm{v}, \mathrm{w}) = (0,0)$ corresponding to the vacuum state. Here, eigenvalues of the Jacobian matrix are given by $\lambda_{\pm} \sim \tilde{P} \pm i$. It can thus be stated that the Andronov-Hopf (AH) bifurcation occurs at around $\tilde{P} = 0$. Supplementary Figure 1 clearly shows that the two nullclines become tangential for large $\tilde{P}$, suggesting that the saddle-node bifurcation on the limit cycle (SNLC) emerges.

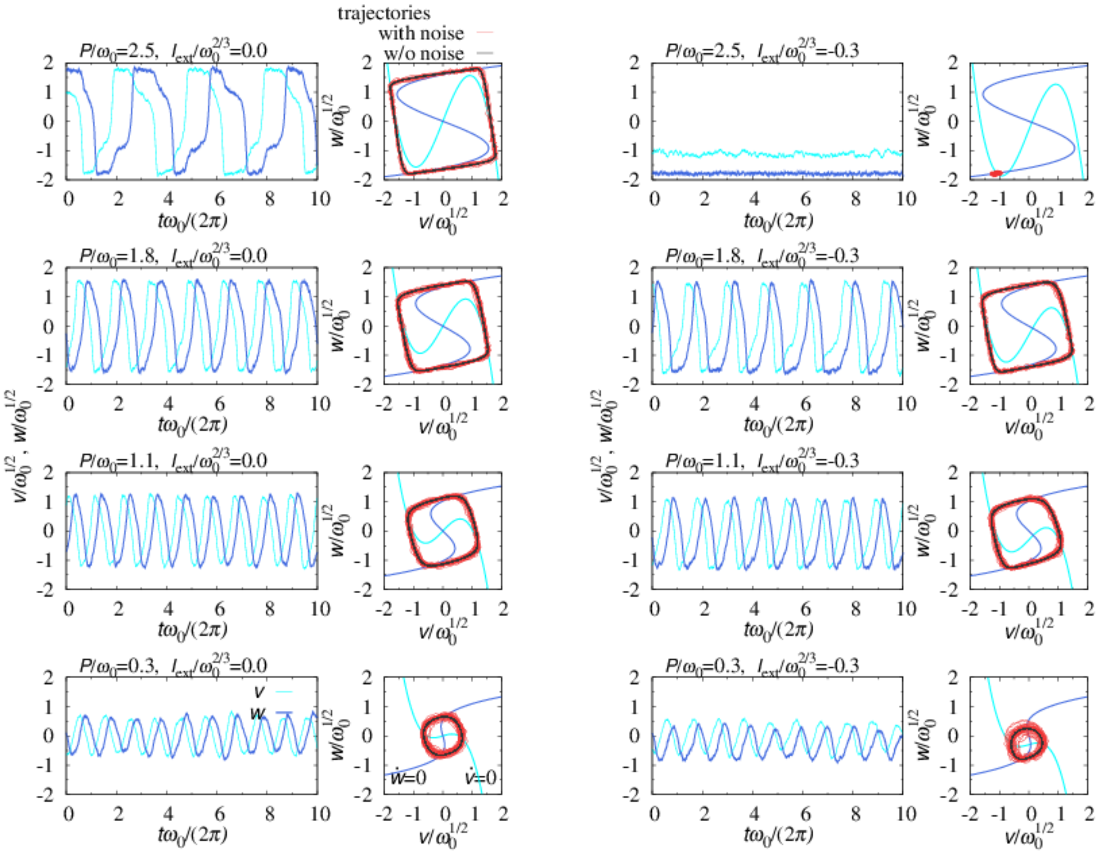

Supplementary Figure 1 Numerical simulation of DOPO neuron dynamics. Time series of DOPO amplitudes, and the trajectories (red) in the v-w space and the nullclines, which are curves satisfying dv/dt=0 (cyan) and dw/dt=0 (blue). For comparison, trajectories calculated without noise terms are also shown (black), which correspond to the orbit of the limit cycles.

Supplementary Figure 1 helps us to discuss the linear stability analysis shown in Method. A linearized form around an equilibrium point $\begin{pmatrix} v \\ w \end{pmatrix} = \begin{pmatrix} v_e \\ w_e \end{pmatrix}$ is given by $\frac{d}{dt}\begin{pmatrix} v \\ w \end{pmatrix} = M \begin{pmatrix} v \\ w \end{pmatrix}$ with $M = \begin{pmatrix} \frac{\partial f(v,w)}{\partial v} & \frac{\partial f(v,w)}{\partial w} \\ \frac{\partial g(v,w)}{\partial v} & \frac{\partial g(v,w)}{\partial w} \end{pmatrix} = \begin{pmatrix} P - 3v_e^2 & -\omega_0 \\ \omega_0 & P - 3w_e^2 \end{pmatrix}$, where function $f(v,w)$ and $g(v,w)$ are the right hand of Supplementary Equations 3 and 4, respectively.

The bottom panels of Supplementary Figure 1 show that there is equilibrium at around $\begin{pmatrix} v_e \\ w_e \end{pmatrix} = \begin{pmatrix} 0 \\ 0 \end{pmatrix}$ for small $P(> 0)$. Around this equilibrium, eigenvalues of M are given by $\lambda = P \pm i\omega_0$, suggesting the AH bifurcation. The top panels of Supplementary Figure 1 show that equilibriums can be found at the tangency points of nullclines around $\begin{pmatrix} v_e \\ w_e \end{pmatrix} \sim \begin{pmatrix} \pm\sqrt{P/3} \\ \mp 2P\sqrt{P}/3\omega_0 \end{pmatrix}$ for large P. Around such equilibriums, two eigenvalues λ can be real and positive values that characterize the SNLC bifurcation. Using numerical calculations, it is possible to precisely estimate equilibriums and corresponding v_e and w_e, and the points where AH or SNLC bifurcations occur can then be evaluated, as shown in Figure 2b in the main text. Numerical simulations also showed that, at the proximity of cross points where these two bifurcation sets merge with each other, other kinds of bifurcations, namely, codimension-two bifurcations, occur (but they are not detailed here). In addition, by simulating the dynamics based on Equations 1 and 2 in the main text, it is also possible to calculate spiking frequency as shown in the color map of Figure 2b and the similar plot with a wider parameter area (Supplementary Figure 2a below). As an example of the codimension two bifurcation, the Bogdanov Takens (BT) bifurcation point is

shown in Supplementary Figure 2b. Note that the numerical simulation needs the higher parameter precision to plot Supplementary Figure 2b than Figure 2b in the main text, suggesting that the experiments require very sensitive techniques for controlling parameters and noises. Experimental observations of various bifurcations is thus a future work. It is confirmed that the two kinds of calculations used here are consistent with each other; namely, the region surrounded by the red (AH) and blue (SNLC) curves coincides with the colored region with finite values of spiking frequency.

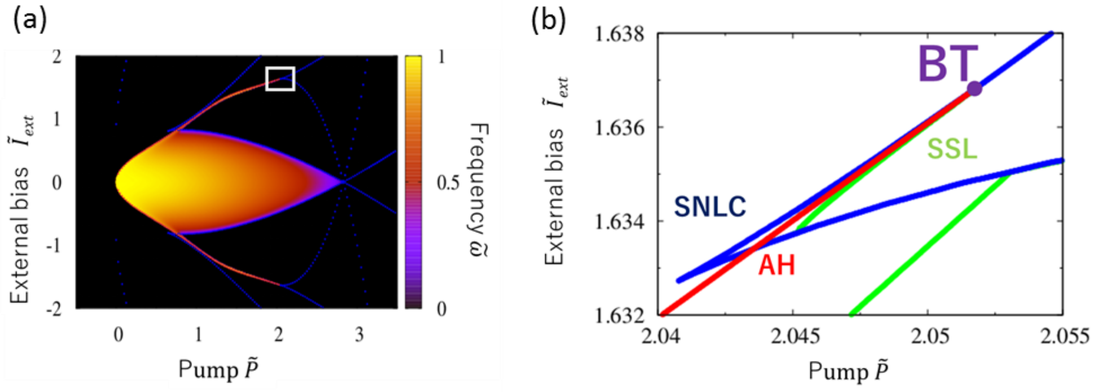

Supplementary Figure 2 Bifurcations of a DOPO neuron. **a** Color map of spiking frequencies as functions of P and I_{ext} calculated by numerical simulations with higher precision than those used in the main text. White square corresponds to the area shown in the panel b. **b** Bifurcation sets in the area shown by the white square in the panel a. Red and blue lines represent points where Andronov-Hopf (AH) bifurcation and saddle-node bifurcation on a limit cycle (SNLC) occur respectively. Green lines are saddle separatrix loop (SSL) bifurcations. The violet point represents Bogdanov Takens (BT) bifurcation point.

Supplementary Note 2 - *Bifurcation model*

Bifurcation models of the present system are analytically discussed hereafter. To capture the essence of the bifurcations found in the present experiments, the noise terms and quadrature components of DOPOs are not taken into account; and thus, variables v and w represent the in-phase components of DOPO amplitudes. At first, the external bias term is not considered. For convenience, a new parameter β is introduced to characterize the property of nonlinear terms. The bifurcation model is thus given by:

$$\frac{dv}{dt} = Pv - (v^2 + \beta w^2)v + J_{vw}w, \tag{7}$$

$$\frac{dw}{dt} = Pw - (\beta v^2 + w^2)w + J_{wv}v. \tag{8}$$

Although this parameter is zero ($\beta = 0$) and is uncontrollable in the present experiments, it helps us to discuss the change of types of bifurcation. It should be noted that some physical systems can be described by the analogical equation to the above with $\beta = 1$, and thus this parameter β makes it possible to clarify the difference between the bifurcation mechanisms of different physical systems.

Hereafter, it is assumed that $J_{vw}J_{wv} < 0$, and only the case of $J_{vw} < 0$ and

$J_{wv} > 0$ is considered. The opposite-sign case is straightforward under the transformation of $v \to -v$. And, w is rescaled as $\sqrt{\alpha}w$, and then the above equations can then be rewritten by using $z = v + iw$ as

$$\frac{dz}{dt} = i\omega_0 z + Pz - \frac{1+\alpha}{2}\left(|z|^2 z - \frac{1-\beta}{4}(z^2 - z^{*2})z^*\right)$$
$$-\frac{1-\alpha}{2}\left(|z|^2 z^* + \frac{1+\beta}{4}(z^2 - z^{*2})z\right), \tag{9}$$

which can be rewritten with $z = \sqrt{R}e^{i\theta}$ and $R = v^2 + w^2/\alpha$ as

$$\frac{d\theta}{dt} = \omega_0 + R\frac{1-\beta}{4}\left(\frac{1+\alpha}{2}\sin 4\theta\ - \frac{1-\alpha}{2}2\sin 2\theta\right), \tag{10}$$

$$\frac{dR}{dt} = 2PR - 2R^2\left[\frac{1+\alpha}{2}\left(\frac{3+\beta}{4} + \frac{1-\beta}{4}\cos 4\theta\right) + \frac{1-\alpha}{2}\cos 2\theta\right]. \tag{11}$$

From the above expressions, it can be easily understand that ω_0 corresponds to the angular frequency of the oscillator at $\beta = 1$, or at the limit of $R \to 0$ with $P \to +0$, where a simple oscillator with $\frac{d\theta}{dt} = \omega_0$ appears. It can thus be expected that the AH bifurcation should occur near these two limits. For example, for $\alpha = \beta = 1$, the normal form of the AH bifurcation is obtained as $\frac{dz}{dt} = i\omega_0 z + Pz - |z|^2 z$. From another viewpoint, two separable variables (θ and R) can be obtained as $\frac{d\theta}{dt} = \omega_0$ and $\frac{dR}{dt} = 2PR - 2R^2$. However, for $\beta = 0$ and $P > 0$, the connection between θ and R induces change of bifurcations from AH to SNLC types. Roughly speaking, the normal form of the saddle-node bifurcation can be derived too; for example,

at $\alpha = 1$ and $\beta = 0$ the form $\frac{d\theta}{dt} = \omega_0 + \frac{R}{4}\sin 4\theta$ can be reduced to $\frac{d\theta}{dt} = \omega_0 + \frac{P}{\sqrt{8}}(-1 + 8\theta^2)$. This point will be discussed again in Supplementary Notes 3. It is thus clear that parameters P and β play important roles in the mechanism of the bifurcations.

Supplementary Note 3 - *Mathematical expressions*

Some mathematical expressions to estimate spiking frequencies and bifurcation points are derived hereafter. Such mathematical expressions make it possible to compare the experimental results with theory and numerical calculations. We should note that in experiments all of the parameters are influenced by various noises, losses, and fluctuations, and thus it is difficult to determine the magnitude of parameters precisely. It is therefore important to use mathematical expressions to discuss the quantitative aspects of experiments.

Spiking frequency ω as a function of optical-pump amplitude P is analytically calculated hereafter. In the main text, parameters α and β are defined as $\alpha = 1$ and $\beta = 0$. Here, $\frac{d\theta}{dt} = \omega_0 + \frac{R}{4}\sin 4\theta$ and $\frac{dR}{dt} = 2PR - \frac{R^2}{2}(3 + \cos 4\theta)$ are obtained. As discussed above, at the limit of $\mathrm{P} \to +0$, a firing frequency ω is equivalent to the basic frequency $\omega_0(\equiv \sqrt{-J_{vw}J_{wv}})$. The experimental and simulation results suggest that the firing rate decreases as P increases as shown in the main text. Averaged R in one cycle of oscillations (during a change of θ in $[-\pi, \pi]$), which corresponds to the static component of R with a condition of $\frac{dR}{dt} = 0,$ is first estimated. It is given by

$R_{\text{ave}} = \frac{2P}{\pi}\int_{-\pi}^{\pi}\frac{d\theta}{3+\cos 4\theta} = \sqrt{2}P$. In the present optical system, it corresponds to that the number of photons ($\propto R = v^2 + w^2$) increases linearly with increasing optical-pump amplitudes P. Neglecting the oscillatory component of R leads $\frac{d\theta}{dt} = \omega_0 + \frac{P}{\sqrt{8}}\sin 4\theta$. The period of the oscillator can then be approximately evaluated as $T = \int_{-\pi}^{\pi}\frac{d\theta}{\omega_0 + \frac{P}{\sqrt{8}}\sin 4\theta} = \frac{2\pi}{\sqrt{\omega_0^2 - P^2/8}}$, and angular frequency ω, corresponding to firing rate, is given as a function of P as follows:

$$\omega(P) = \omega_0\sqrt{1 - \frac{P^2}{8\omega_0^2}}. \tag{12}$$

Imposing the limit of $P \to +0$ gives basic frequency ω_0 as mentioned above, suggesting that the AH bifurcation (class II neuron) can be found near $P \sim 0$. At $P = P_{SNLC} \equiv \sqrt{8}\omega_0$, the frequency gradually reaches zero, suggesting the SNLC bifurcation (the class I neuron). Point P_{SNLC} is exactly the same as the analytically obtained expression of the bifurcation point as mentioned below. Near $P \sim P_{SNLC}$, four points of θ with $\frac{1}{4}(\frac{\pi}{2} + 2n\pi)$ with $n = 0,1,2,3$ satisfy $\frac{d\theta}{dt} \sim 0$, suggesting that θ stays near these points in almost all of one cycle. The period of the saddle node bifurcations is then obtained as $T \sim \frac{4}{\omega_0}\int_{-\infty}^{\infty}\frac{d\theta}{1 - \frac{P}{P_{SLNC}} + \frac{8P}{P_{SLNC}}\theta^2}$. Finally, $\omega_{P \sim P_{SNLC}}(P) = \omega_0\sqrt{\frac{2P}{P_{SNLC}}}\sqrt{1 - \frac{P}{P_{SNLC}}}$, which is equivalent with the above expression near $P \sim P_{SNLC}$, is obtianed. Note

that there are four points satisfying $\frac{d\theta}{dt} \sim 0$ for $\alpha = 1$ and $I_{ext} = 0$ because of two kinds of symmetry, sign-inversion $[(v, w) \to (-v, -w)]$ and v-w-inversion $[(v, w) \to (w, v)]$ symmetry: For $\alpha \neq 1$ the latter is broken, and for $I_{ext} \neq 0$ both are broken.

The above approximated expressions of $\omega(P)$ is compared with the numerical calculations. Supplementary Figure 3 shows spiking frequencies as a function of optical-pump amplitude P. Near both end points ($P \sim 0$ or $P \sim P_{SNLC}$), Supplementary Equation 12 agrees well with the numerical results. It should be noted that the numerical simulations have been done with a small enough dt to reproduce the continuous limit, even though discreteness of dt induces quantitative deviations. However, simulations with a larger dt and experiments with discrete timing of feedback signals show qualitatively similar behavior. We should note that the nonlinear terms such as $Pv - v^3$, which are optically implemented in nonlinear optics, are perfectly continuous in experiments, even though the linear terms implemented by the measurement feedback method are discrete.

Mathematical expressions of the bifurcation points are shown next. Without external fields $\tilde{I}_{ext} = 0$, the P values where the bifurcation occurs

can be calculated analytically. As mentioned above, the $P \to 0$ limit reproduces the normal form of the AH bifurcation. From this point, the bifurcation point is obtained as $\tilde{P}_{\mathrm{AH}} = 0$. For the SNLC bifurcations, the points where two nullclines have tangency can be calculated. Bifurcation point $\tilde{P}_{SNLC}$ can thus be easily calculated by solving a cubic equation $4\alpha\left(4+\tilde{P}^2\right)^3 = 27\tilde{P}^4(\alpha+1)^2$, which yields

$$\tilde{P}_{SNLC} = \sqrt{\frac{1}{2} + \frac{9}{4}A + \frac{3}{2}\sqrt{(-14+9A)(2+A)}\cos\Psi}, \tag{13}$$

where $\Psi = \frac{1}{3}\mathrm{acos}\left(\frac{(27A^2-36A-52)}{(-14+9A)^{\frac{3}{2}}(2+A)^{\frac{1}{2}}}\right) + \frac{4\pi}{3}$ and $A = \alpha + \frac{1}{\alpha}$. For $\alpha = 1$, $\tilde{P}_{SNLC} = \sqrt{8}$ is obtained as mentioned above.

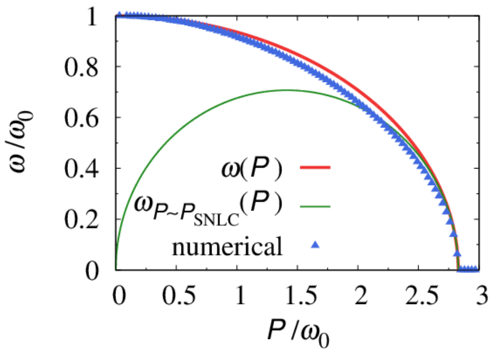

Supplementary Figure 3 Comparison between numerical calculations and analytical expressions. Spiking frequency as a function of pump amplitude P calculated by the numerical simulations (blue triangles) and analytical forms (red and green lines).

Supplementary Note 4 - *Spontaneous modification of spiking frequency*

Connected spiking DOPO neurons are discussed hereafter. The following parameters are set as $\alpha = 1$, $\beta = 0$, and $I_{\mathrm{ext}} = 0$ for simplicity. The dynamics of DOPOs is described by Equations 1 and 2 in the main text. Here, the matrix J_{ij} describes connections of DOPO neurons and is an N by N matrix, where N is the number of DOPO neurons (i.e., the number of DOPO pulses is $2N$).

The following standard Kuramoto model[4,5] is considered to explain one of the essential points of the synchronization of the DOPO neurons:

$$\frac{d\theta_i}{dt} = \omega_i - J_K \sum_j \sin(\theta_i - \theta_j), \tag{14}$$

where node-dependent ω_i is assumed to be a natural distribution with a variance σ_ω. In this well-known model, the synchronization can be understood from the analogy to the phase transition with order parameter $re^{i\psi} = \frac{1}{N}\sum_j e^{i\theta_j}$.

The analogy between the DOPO neuron model and the Kuramoto model, as well as their differences, are discussed hereafter. Here, parameters in terms of connections are set as $\gamma = \gamma'(\equiv J_K)$ and $J_{ij} = 1 - \delta_{ij}$, where J_{ij} represents that the DOPO neurons have connections on a complete graph

structure. By assuming that i-dependence on R_i is negligibly small, Supplementary Equations 1 and 2 are rewritten as

$$\frac{d\theta_i}{dt} = \omega_0 + \frac{R}{4}\sin 4\theta_i - J_K \sum_j \sin(\theta_i - \theta_j), \tag{15}$$

$$\frac{dR}{dt} = 2P_i R - \frac{R^2}{2}(\cos 4\theta_i + 3) + 2J_K R \sum_{j\neq i} \cos(\theta_i - \theta_j). \tag{16}$$

As mentioned above, the frequency of an oscillator can be roughly evaluated as $\omega_i(P_i) = \omega_0\sqrt{1 - \frac{P_i^2}{8\omega_0^2}}$. Finally, the effective Kuramoto model is given by

$$\frac{d\theta_i}{dt} = \omega_i(P_i) - J_K \sum_j \sin(\theta_i - \theta_j). \tag{17}$$

As discussed later, it should be noted that the frequencies $\omega_i(P_i)$ include the effects of the synchronization via pump renormalization.

Pump renormalization effects, which are characteristic of the synchronization of the DOPO neurons, are discussed next. From Supplementary Equation 16 it can be inferred that optical-pump amplitude should be renormalized as $\tilde{P}_i' = \tilde{P}_i + \tilde{J}_K \sum_{j\neq i} \cos(\theta_i - \theta_j)$. By using the order parameter of the Kuramoto model defined as $re^{i\psi} = \frac{1}{N}\sum_j e^{i\theta_j}$, renormalized pump amplitude is obtained as $\tilde{P}_i' = \tilde{P}_i + r(N-1)\tilde{J}_K$. This expression suggests that the synchronization causes an effective change in optical-pump amplitude; thus, at the network level, the synchronization induces crossover between AH and SNLC bifurcations. This pump renormalization is the

mechanism of the large shift of the spiking frequency after synchronization as discussed in the main text.

To confirm the mechanism above, dynamics of coupled 100 DOPO neurons was numerically simulated. Supplementary Figure 4 shows that the change in spiking frequencies is caused by the synchronization. The left panel shows frequencies as functions of an interaction strength J_K and also corresponding $P'(=\tilde{P}_i + r(N-1)\tilde{J}_K)$, where the change in frequencies of the single DOPO neuron as a function of the original (not renormalized) pump amplitude P is also shown for comparison. The right panel shows histograms of frequencies for NJ_K/ω_0= 0, 0.1, 0.4,…,1.6.

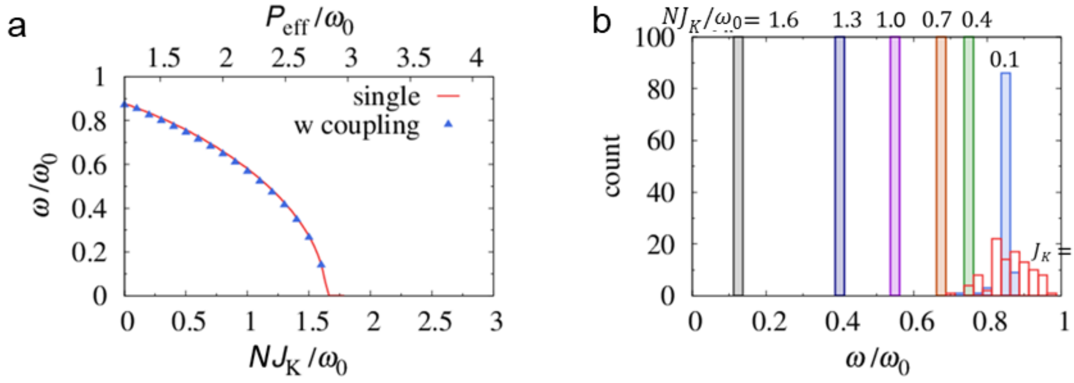

Supplementary Figure 4 Renormalized firing rate. **a** Firing rate $\tilde{\omega}$ (on average over the neuronal indices) as a function of $\tilde{J}_K$ or $\tilde{P}$. For comparison, spiking frequencies for a single DOPO neuron obtained by numerical simulation are also shown by a red line. **b** Histogram of $\tilde{\omega}$ for NJ_K/ω_0=0, 0.1,0.4,…,1.6.

Supplementary Note 5 - *Spiking dynamics depending on order parameter*

As discussed in the main text, the spiking mode of clustered DOPO neurons can be changed according to order parameter of the synchronization. Here, to show where the changes in spiking mode and spiking frequency occur, additional figures plotted from the data in Figure 3 in the main text are depicted. As for Supplementary Figure 5b, the spiking frequencies are briefly estimated from the transit time of each fourth quadrant in the v-w plane. As for Supplementary Figure 5c, the time evolutions of the phases of sampled neurons of four clusters (A, B, C, D) show stepwise changes indicating the class-I neuron, when the order parameter is increased as shown in Supplementary Figure 5d. On the other hand, the neuron in the cluster A (index = 1) shows fast and continuous behavior when the order parameter is decreased by the phase-mismatch of four clusters. Since the neurons in the cluster A are assigned to lower pump amplitudes than the others, the effective pump amplitude could be shifted to the class-II region by the pump renormalization according to the order parameter. In domains where the estimated spike frequency is over 4 kHz, as shown in

Supplementary Figure 5b, the spiking mode changes from the class-I to class-II neuron.

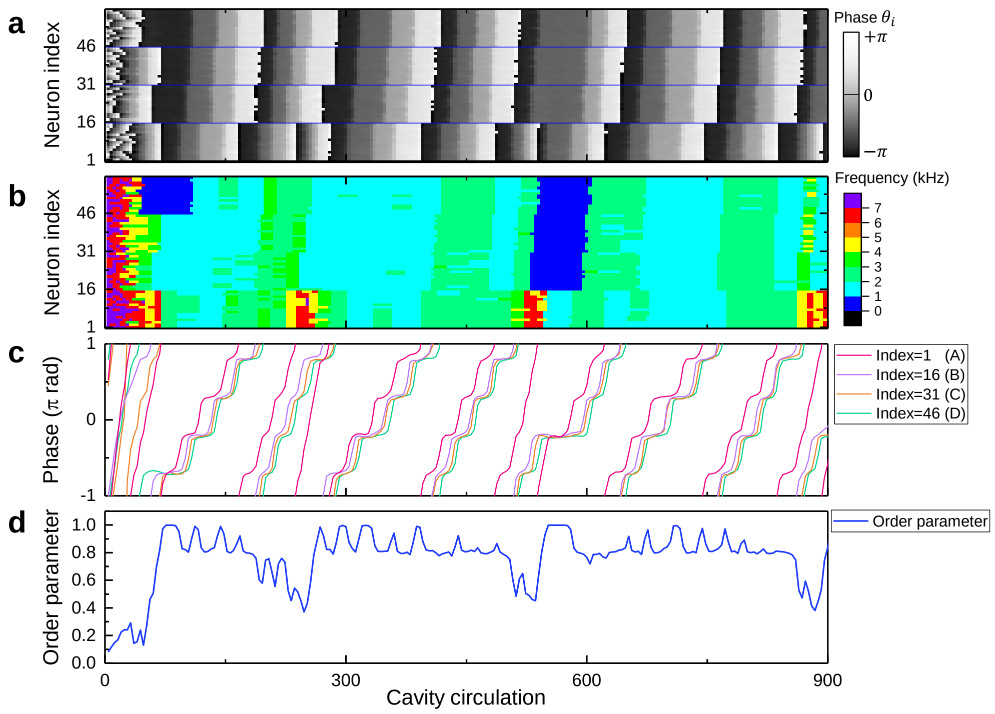

Supplementary Figure 5 Synchronized behavior of clustered DOPO neurons. **a** Time evolutions of phases of the DOPO neurons. **b** Spiking frequencies estimated from the transit time of each fourth quadrant in the v-w plane. **c** Phases of sampled neurons in each cluster. **d** Order parameter for all 60 neurons

Supplementary Note 6 - ***Additional data of synchronization experiment***

As discussed in the main text, synchronization phenomena of networked DOPO neurons that indicate the pump renormalization effects mentioned in section S-D was observed in experiments. Figure 3a shows network of 60 DOPO neurons, in which 15 neurons form an all-to-all connected cluster, and four such clusters were sparsely connected. This network structure was encoded into connections of both the v- and w-DOPOs (that is, $\gamma = \gamma' \equiv J_k$). Four independent sets of such ensembles consisting of 60 DOPO neurons with different coupling strengths ($\widetilde{J_k}$ = 0, 0.025, 0.05, and 0.075) were implemented, external field was turned off $I_{\text{ext}} = 0$, and uniform (i-independent) coupling of $|J_{vw}| = |J_{wv}|$ was used. Optical-pump amplitude P_i was set to be i-dependent to generate the distribution of $\omega_i(P_i)$ and was assigned to the four 15-neuron clusters labeled A to D in descending order of firing rate. Figure 3b shows the distribution of measured firing rates of 60 DOPO neurons. Without coupling (J_k= 0), the firing rates are widely spread according to applied pump P_i. As J_k increases, mean and variance of firing rate are decreased. Supplementary Figure 6a and 6b shows time evolutions of the phase θ_i of ith DOPO neuron and order parameter r for

each cluster (N = 15) and for all neurons (N = 60), respectively. With a weak coupling at $\tilde{J}_k$ = 0.025, coupled DOPO neurons show obvious synchronization in each 15-neuron cluster; however, the total order parameter still takes low values because the four synchronized clusters have different firing rates. At $\tilde{J}_k$ = 0.050, the order parameter averaged over all neurons increases almost periodically, and at $\tilde{J}_k$=0.075, the four clusters achieve intermittent synchronization.

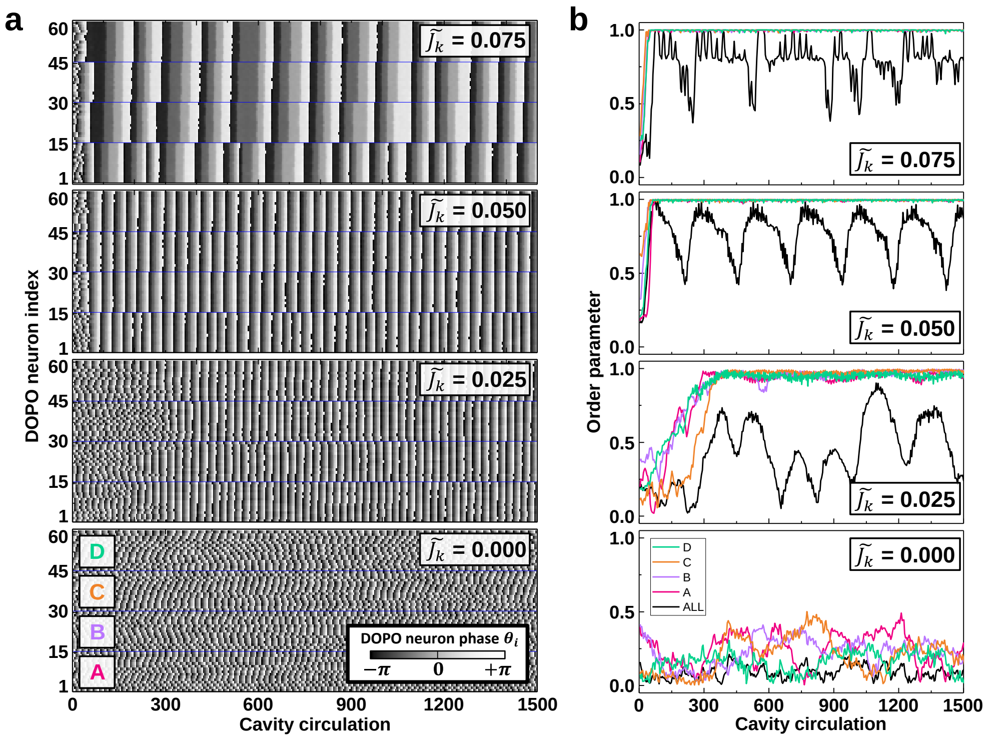

Supplementary Figure 6 Synchronization experiment for clustered Kuramoto models. **a** Time evolutions of phase of the ith DOPO neuron. **b** Time evolutions of the order parameter for each cluster and for all 60 neurons.

Synchronization like the (not clustered) Kuramoto model was also observed, when all neurons were connected in a single cluster. Spiking frequencies of 60 connected neurons had similar distribution to those found in the four cluster experiment by applying the same pump amplitude P_i. By increasing the coupling strength $\widetilde{J_k}$, the phase locking of all neurons and the high order parameter $r \sim 1$ were found as shown in Supplementary Figure 7. Since the number of connections of a single 60-neuron cluster is larger than those of four 15-neuron clusters, the fully-connected 60 neurons achieved synchronization with smaller coupling strength $\widetilde{J_k}$.

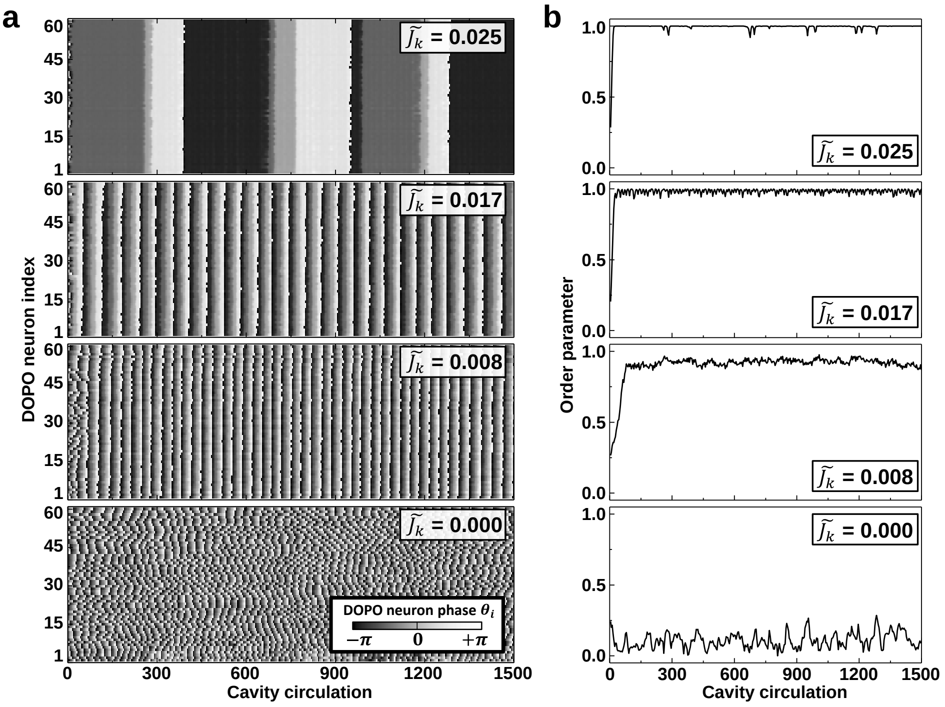

Supplementary Figure 7 Synchronization experiment of a single Kuramoto model. **a** Time evolutions of phases of the DOPO neurons. **b** Time evolutions of the order parameter for all 60 neurons.

Supplementary Note 7 - *Ising model solver*

The use of the network of DOPO neurons as an Ising model solver, where the goal is to minimize the Ising energy, is discussed hereafter. Spin-spin interaction of the Ising model is implemented with the coupling matrix J_{ij} where we set $\gamma = -J_K$ and $\gamma' = 0$. To take account of the mechanism of the control of the firing rates, the effective "order" parameter is defined as $r_i e^{i\psi_i} = \sum_j \sqrt{\frac{R_j}{R_i}} J_{ij} e^{i\theta_j}$ with $C_i + i\,S_i = r_i e^{i(\psi_i - \theta_i)}$, where $C_i = \sum_j \sqrt{\frac{R_j}{R_i}} J_{ij} \cos(\theta_j - \theta_i)$ and $S_i = \sum_j \sqrt{\frac{R_j}{R_i}} J_{ij} \sin(\theta_j - \theta_i)$. Under the ideal conditions $R_i \sim R_j$ and $\theta_j - \theta_i \sim \{0, \pi\}$, the real part C_i reduces to $\sum_j J_{ij} \cos(\theta_j - \theta_i)$ with $\cos(\theta_j - \theta_i) = \pm 1$, which is equivalent with the local energy $E_{\mathrm{loc},i}$. Note that the present DOPO neuron has the sign-inversion symmetry of $(v, w) \to (-v, -w)$, and as a result, two symmetric equilibrium points with a phase difference of π usually appear in the v-w plane. This result suggests that condition $\theta_j - \theta_i \sim \{0, \pi\}$ is expected to be satisfied in a large P region ($P \geq P_{SN}$), or to be satisfied for a long time in a class-I region (at all times except during firing).

With C_i and S_i, the above equations can be rewritten as

$$\frac{d\theta_i}{dt} = \omega_0' + \frac{R_i}{4}\sin 4\theta_i + \frac{J_K}{2}(S_i \cos 2\theta_i + C_i \sin 2\theta_i), \tag{18}$$

$$\frac{dR_i}{dt} = 2P_i' R_i - \frac{R_i^2}{2}(\cos 4\theta_i + 3) - J_K R_i (C_i \cos 2\theta_i - S_i \sin 2\theta_i), \tag{19}$$

where parameters frequency and pump are renormalized as $\omega_0' = \omega_0 - \frac{J_K S_i}{2}$ and $P' = P - \frac{J_K C_i}{2}$. The renormalization of these two parameters yields the change in firing rates. Ideally, energetically stable neurons have large P', suggesting positive correlation of firing rate and local energy. In addition, $\cos 2\theta_i$ and $\sin 2\theta_i$ terms change the saddle-node bifurcation points P_{SNLC} (see α dependence of P_{SNLC} in Supplementary Notes 3). Change in relative value P/P_{SNLC} causes change of firing rate because firing frequency largely changes near $P = P_{SNLC}$. Supplementary Figure 8 confirms the above discussions in the same way as Supplementary Figure 4. Note that deviation from ideal firing rate may become quite large, but the essential qualitative features can be surely captured by the above discussions.

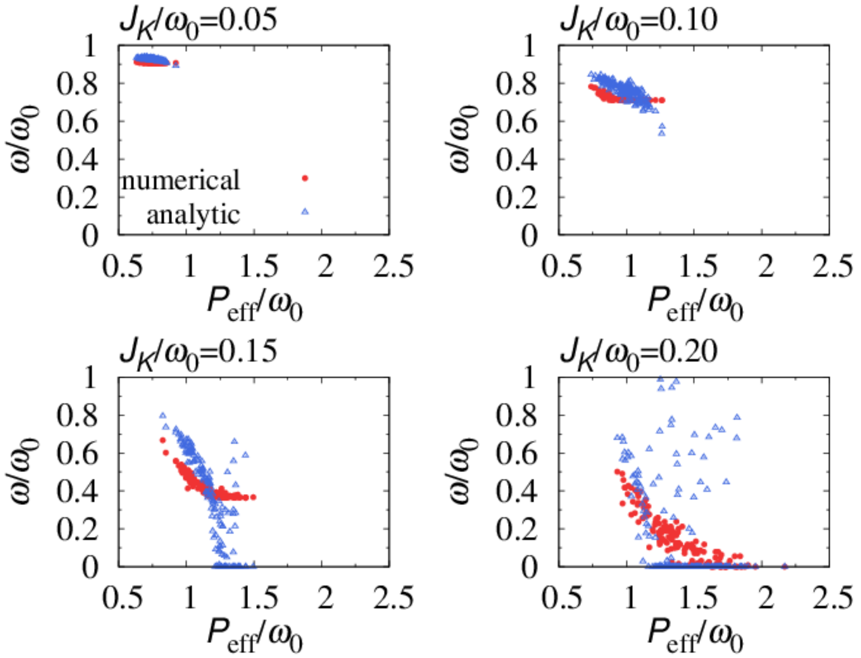

Supplementary Figure 8 Renormalized firing rate. Numerically obtained firing rates for the Ising solver with fixed pump amplitude, and firing rates analytically estimated considering renormalization of P and ω_0, which are obtained with the aid of the numerical simulations calculating the average values of C_{i} and S_{i}.

As discussed above, the DOPO neurons with higher (lower) local Ising energy showed higher (lower) firing rates, and a positive correlation between firing count and local energy was observed as shown in Figure 4c in the main text. Such a selective spin-flip mechanism could help the optimization procedure of the Ising model. Furthermore, to take advantage of the characteristics of the class-II and class-I neurons, pump amplitude was gradually increased as shown in Supplementary Figure 9a. At the early stage of the computation, the Ising energy was rapidly decreased as shown in

Figure 4b in the main text. The DOPO neurons behaved as the class-II mode due to the lower pump amplitude, and the spiking frequency near ω_0 was used for the rapid search process in the early stage. To decrease the Ising energy effectively, the speed of pump increasing should be much slower than spike firing time defined by $1/\omega_0$. In the final stage, on the other hand, the spin configuration of the Ising model was frozen when pump amplitude reached the maximum of the spiking region. To obtain the final solution at the lowest energy, pump amplitude should be increased gradually in the class-I region to achieve the gradual freezing-out at the end of the computation. Supplementary Figure 9b shows the time evolution of the success probability of the ground state search in 100 trials. The success probability of the final solution was slightly decreased from that of solutions during the computation. This result means that the spin configurations were frozen at slightly higher energy state, and the success probability of the final solution could be improved by slowing down the pump increasing speed.

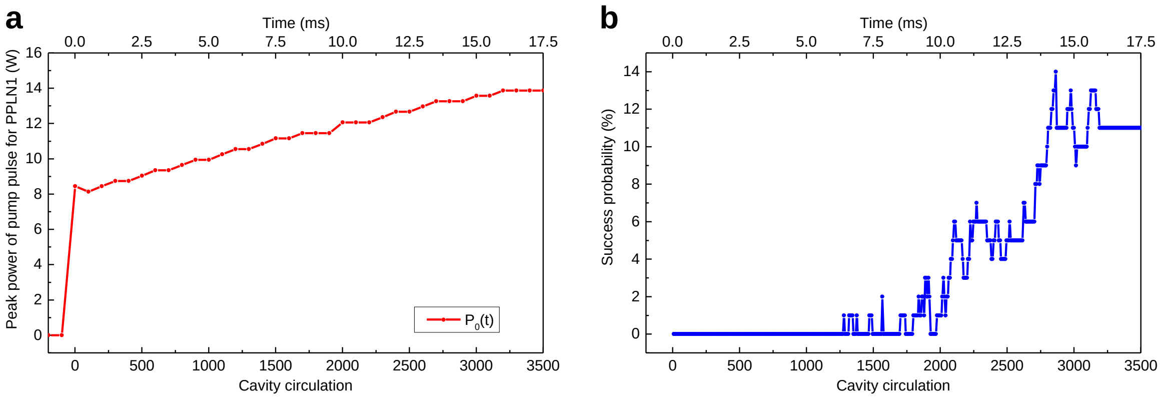

Supplementary Figure 9 Pump schedule and success probability when solving a 150-node Ising problem. **a** Measured peak power of pump pulse for the SHG process in the case of the PPLN1. The peak power was approximately proportional to pump amplitude for the PSA process in the case of the PPLN2. **b** Time evolutions of the success probability evaluated from time evolutions of Ising energy in 100 trials.

Success probability with the DOPO spiking neural network (DOPO-SNN) and the coherent Ising machine (CIM) in our previous work[6] for the same instance are compared as follows. Supplementary Figure 10 shows the histogram of the Ising energy of obtained solutions, where computation times were 17.5 ms and 5 ms for the DOPO-SNN and the CIM, respectively. The CIM used a pitchfork bifurcation of networked DOPOs to find the optimal phase configuration to minimize the total optical loss in the network. The spin configuration of the Ising model could be obtained from the phase configuration of the DOPOs. Since the pitchfork bifurcation process was very fast, the CIM could find the solutions in 5 ms, which corresponds to 1000

cavity circulations. Although the CIM showed good performance for many instances in previous works, success probability for the ground state search for this instance was 0.8%. Because it was difficult to change the phase configuration of DOPOs after the pitchfork bifurcation, the solutions of the CIM could be trapped in the local minimum states for this instance. On the other hand, the DOPO-SNN could find the ground state with a high success probability of 11% in computation time of 17.5 ms, which corresponds to 3500 cavity circulations. By introducing the spiking dynamics, the phase configuration of the DOPO neurons could continue to change until the solution escaped from the local minimum state and reached the ground state. With the assistance of the selective spin-flip mechanism discussed above, the DOPO-SNN showed better performance than the CIM in terms of the time-to-solution for this hard instance. It is useful for comparison to consider the time-to-solution with 99% success probability which is given as $t_{99} = T \log(1-0.99) / \log(1-P_s)$, where T is the computation time and P_s is the success probability[6]. CIM and the proposed spiking dynamics have t_{99} = 2867 ms and 692 ms, respectively. Thus, the time-to-solution with 99% success probability is reduced by a factor of four by introducing the spiking dynamics.

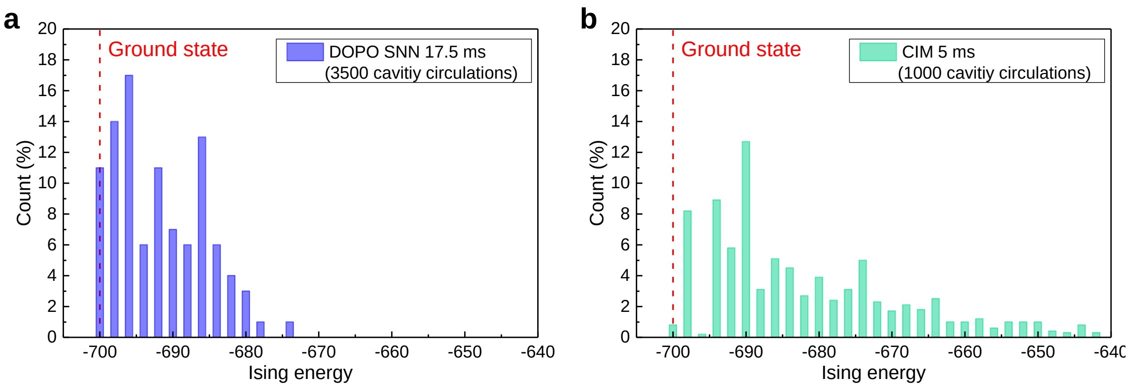

Supplementary Figure 10 Histogram of Ising energy in the solution for a 150-node Ising problem. **a** Histogram of 100 solutions with the DOPO-SNN for computation time of 17.5 ms for each trial. **b** Histogram of 1000 solutions with the CIM for computation time of 5 ms for each trial.

It should be noted that the time scale of spiking frequency, ω_0, restricts the circulation steps (N_{step}) of the DOPO-SNN scheme; i.e., $N_{step}\omega_0 \gg 1$. Namely, a sufficient number of spikes is required during all circulations. Here, ω_0~1/20 (with a unit of [spikes/steps]) with N_{step}=3500 was used. This ω_0 is determined from experimental parameter J_{vw}. Note that an important parameter regarding the Ising problem solver (see Figure 4 in the main text) is the ratio between spiking frequency (J_{vw}) and Ising coupling (J_K), corresponding to $\widetilde{J_k}$ (= J_K/ω_0). Controlling parameter $\widetilde{J_k}$ is now strictly limited due to the performance of the FPGA; namely, J_{vw} and J_K should be 8 bit integers. It is expected that the performance of the FPGA can be improved in the future, and furthermore, v-w coupling J_{vw} can be replaced by

direct optical coupling; and then, the ratio of J_{vw} and J_K can be tuned widely. We thus believe that the current limitation on number of circulation steps can be relaxed in the future work.

Supplementary References:

1. Drummond, P. D., McNeil, K. J. & Walls, D. F. Non-equilibrium transitions in sub/second harmonic generation. *Optica Acta: International Journal of Optics* **27**, 3321–335 (1980).
2. Haribara, Y., Yamamoto, Y., Kawarabayashi, K. I. & Utsunomiya, S. A coherent Ising machine for MAX-CUT problems: performance evaluation against semidefinite programming relaxation and simulated annealing. *Encyclopedia of Spectroscopy and Spectrometry*, 3rd Edition (Elsevier, 2016); http://arxiv.org/abs/1501.07030 (2015).
3. Kinsler, P. and Drummond, P. D. Quantum dynamics of the parametric oscillator. *Phys. Rev. A* **43**, 6194 (1991).
4. Kuramoto, Y. Self-entrainment of a population of coupled non-linear oscillators. International Symposium on Mathematical Problems in Theoretical Physics. *Lecture Notes in Physics*, **39**. Springer, Berlin, Heidelberg (1975).
5. Acebrón, J. A., Bonilla, L. L., Vicente, C. J. P., Ritort, F. & Spigler, R. The Kuramoto model: A simple paradigm for synchronization phenomena. *Rev. Mod. Phys.* **77**, 137 (2005).
6. Hamerly, R. et al. Experimental investigation of performance differences between coherent Ising machines and a quantum annealer. *Sci. Adv.* **5**, eaau0823 (2019).
